# Association of Distance, Region, and Insurance With Advanced Colon Cancer at Initial Diagnosis

**DOI:** 10.1001/jamanetworkopen.2022.29954

**Published:** 2022-09-02

**Authors:** Nathan R. Brand, Anya L. Greenberg, Sy H. Chiou, Mohamed Adam, Ankit Sarin

**Affiliations:** 1 Department of Surgery, University of California San Francisco; 2 Department of Mathematical Sciences, University of Texas at Dallas, Richardson

## Abstract

This cross-sectional study examines factors associated with the stage at which colon cancer is diagnosed.

## Introduction

In the US, more than 100 000 new colon cancer diagnoses and 35 000 colon cancer deaths are estimated to occur each year.^1^ Less than 40% of patients with new diagnoses will have early-stage disease, which has a 5-year overall survival as high as 90%.^2^ There is a need to identify interventions to promote the early diagnosis of colon cancer.

## Methods

In a cross-sectional design, we analyzed the National Cancer Database on January 11, 2022, using R, version 4.1.2 (R Foundation for Statistical Computing). We included patients aged 18 years or older from 2010 to 2017 whose primary cancer diagnosis was colon cancer; rectal cancer, multiple cancer diagnoses, and missing data for the variables analyzed were excluded. A secondary analysis was performed to identify variables associated with advanced pathologic stage. This analysis excluded patients who did not receive surgical intervention, received neoadjuvant therapy, or received surgical intervention on multiple sites during their index operation. Data analysis was conducted from February 15 to April 15, 2022. Significance was set at .05, and all analyses were 2-sided. This report follows the STROBE reporting guideline for observational studies and was approved by the University of California San Francisco institutional review board with a waiver of patient consent due to the retrospective nature of the study.

## Results

Demographic data for the 208 085 patients with clinical-stage colon cancer are reported in the Table. Multivariable regression adjusting for all variables showed that, compared with patients who lived less than 20.2 km (to convert kilometers to miles, divide by 1.6) from their medical facility, those traveling a greater distance had increasing odds of presenting with advanced clinical-stage disease: 20.2 to 80.0 km (odds ratio [OR], 1.11; 95% CI, 1.09-1.14), 81.0 to 400.0 km (OR, 1.39; 95% CI, 1.34-1.44), and more than 400.0 km (OR 1.78, 1.62-1.94).

**Table.** Demographic Characteristics of Patients With Clinical-Stage Colon Cancer

Characteristic	No. of patients (%) (N = 208 085)
Clinical stage	
Stages 1/2	102 058 (49.0)
Stages 3/4	106 027 (51.0)
Sex	
Female	102 869 (49.4)
Male	105 216 (50.6)
Age, y	
18-49	20 216 (9.7)
50-59	47 125 (22.6)
60-69	55 069 (26.5)
70-79	46 638 (22.4)
≥80	39 037 (18.8)
Race^a^	
Asian	7029 (3.4)
Black	29 287 (14.1)
Hispanic/Spanish	12 489 (6.0)
White	157 369 (75.6)
Other^b^	1911 (1.0)
Income quartile	
1	39 226 (18.9)
2	54 743 (26.3)
3	49 104 (23.6)
4	65 012 (31.2)
Payer	
Commercial	76 639 (36.8)
Medicaid	14 807 (7.1)
Medicare	105 975 (50.9)
Other government insurance	2117 (1.0)
Uninsured	8547 (4.1)
Charlson Comorbidity Index	
0	147 338 (70.8)
1	41 587 (20.0)
2	12 160 (5.8)
≥3	7000 (3.4)
Distance from reporting facility, miles	
0-12.49	133 498 (64.2)
12.5-49.9	58 241 (28.0)
50-249.9	14 233 (6.8)
≥250	2113 (1.0)
Facility location	
South Atlantic	46 931 (22.6)
East North Central	37 227 (17.9)
East South Central	14 239 (6.8)
Middle Atlantic	33 997 (16.3)
Mountain	8103 (3.9)
New England	12 980 (6.2)
Pacific	22 856 (11.0)
West North Central	13 650 (6.6)
West South Central	18 102 (8.7)

^a^ Race was included given the importance of including race in scientific studies, even if there are no significant associations.

^b^ National Cancer Database code referring to all races not specified.

Among 238 309 patients who underwent surgical resection, multivariable regression analysis adjusted for all variables showed that having no insurance (OR, 1.33; 95% CI, 1.27-1.39) or having only Medicaid (OR, 1.22; 95% CI, 1.18-1.27) was associated with advanced pathologic stage after adjusting for all variables. When the analysis was repeated with an interaction term between distance traveled and region of the US, being 81 to 400 km from a treating center and living in the Northeast, Mountain, or Central US were associated with an increased risk of advanced pathologic disease compared with those living in the South Atlantic region (Figure).

**Figure.**
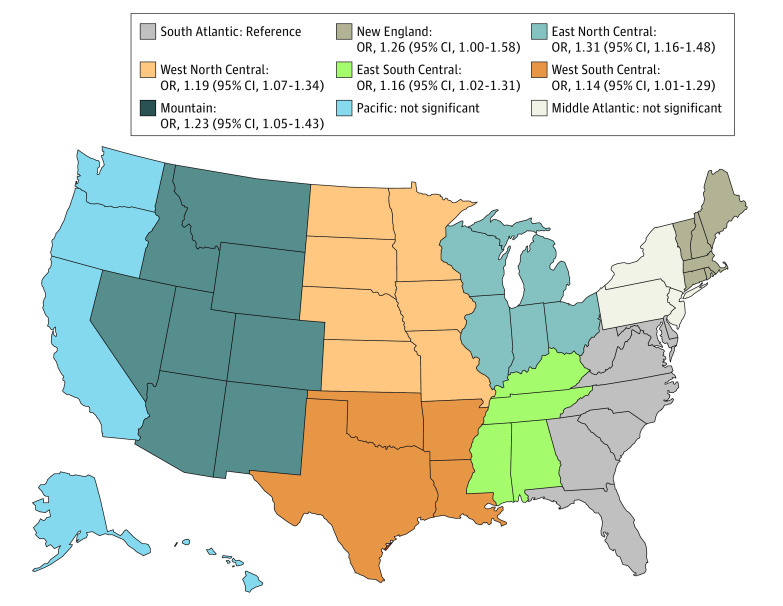
Distance and Advanced Pathologic Stage OR indicates odds ratio.

## Discussion

In our study, distance from the treating facility was associated with advanced-stage colon cancer. This finding was not uniform and patients living in the Mountain, Central, and Northeast regions had a higher odds of advanced-stage disease if living 81 to 400 km from their treating facility compared with patients living in the Southeast.

We also identified insurance status as a risk factor for diagnosis of advanced-stage colon cancer. These findings support previous studies that have identified marginal insurance as an important risk factor for both poor overall survival and advanced-stage disease at presentation.^3,4,5^ The primary limitation of our study is that income and distance were calculated at the zip code rather than patient level, which may create bias.

The findings of this study suggest that distance from the treating facility and insurance status are risk factors for the diagnosis of advanced-stage colon cancer among patients in the US. These findings are important as more rural hospitals are closing and care becomes more regionalized at high-volume centers. Interventions to provide transportation may be useful in the Mountain, Central, and Northeast regions, where patients living far from hospitals have greater risk for diagnosis of advanced-stage disease.
